# Prevalences of inherited red blood cell disorders in pregnant women of different ethnicities living along the Thailand-Myanmar border

**DOI:** 10.12688/wellcomeopenres.12338.2

**Published:** 2017-11-02

**Authors:** Germana Bancone, Mary Ellen Gilder, Nongnud Chowwiwat, Gornpan Gornsawun, Elsi Win, Win Win Cho, Eh Moo, Aung Myat Min, Prakaykaew Charunwatthana, Verena I. Carrara, Nicholas J. White, Francois Nosten, Rose McGready

**Affiliations:** 1 Centre for Tropical Medicine and Global Health, Nuffield Department of Medicine, University of Oxford, Oxford, OX3 7BN, UK; 2 Shoklo Malaria Research Unit, Mahidol–Oxford Tropical Medicine Research Unit, Faculty of Tropical Medicine, , Mahidol University, Mae Sot, Thailand; 3 Department of Clinical Tropical Medicine, Faculty of Tropical Medicine, Mahidol University, Bangkok, Thailand; 4 Mahidol–Oxford Tropical Medicine Research Unit, Faculty of Tropical Medicine, Mahidol University, Bangkok, Thailand

**Keywords:** G6PD deficiency, hemoglobinopathies, blood groups, ethnic groups, Thailand-Myanmar border, anemia, pregnancy

## Abstract

**Background**: Inherited red blood cell disorders are prevalent in populations living in malaria endemic areas; G6PD deficiency is associated with oxidant-induced haemolysis and abnormal haemoglobin variants may cause chronic anaemia. In pregnant women, microcytic anaemia caused by haemoglobinopathies mimics iron deficiency, complicating diagnosis and treatment. Anaemia during pregnancy is associated with morbidity and mortality. The aim of this study was to characterise the prevalence of G6PD deficiency and haemoglobinopathies  among the pregnant population living along the Thailand-Myanmar border. Pregnant women attending antenatal clinics in this area belong to several distinct ethnic groups.

**Methods**: Data were available for 13,520 women attending antenatal care between July 2012 and September 2016. Screening for G6PD deficiency was done by fluorescent spot test routinely. G6PD genotyping and quantitative phenotyping by spectrophotometry were analysed in a subsample of women. Haemoglobin variants were diagnosed by HPLC or capillary electrophoresis and molecular methods. The prevalence and distribution of inherited red blood cell disorders was analysed with respect to ethnicity.

**Results**: G6PD deficiency was common, especially in the Sgaw Karen ethnic group, in whom the G6PD Mahidol variant allele frequency was 20.7%. Quantitative G6PD phenotyping showed that 60.5% of heterozygous women had an intermediate enzymatic activity between 30% and 70% of the population median. HbE, beta-thalassaemia trait and Hb Constant Spring were found overall in 15.6% of women. Only 45.2% of women with low percentage of HbA _2_ were carriers of mutations on the alpha globin genes.

**Conclusions**: Distribution of G6PD and haemoglobin variants varied among the different ethnic groups, but the prevalence was generally high throughout the cohort. These findings encourage the implementation of an extended program of information and genetic counselling to women of reproductive age and will help inform future studies and current clinical management of anaemia in the pregnant population in this region.

## Introduction

Inherited red cells disorders (IRD), such as haemoglobinopathies and G6PD deficient variants, are common in South-East Asian populations living in area of past and present malaria transmission ( Fucharoen & Winichagoon, 2012; Howes *et al.*, 2012; Williams & Weatherall, 2012). Characterization of IRD is important for understanding the causes of anaemia in the population, especially in women during pregnancy when the distinction between physiologic and pathologic anaemia becomes paramount. Outcomes for inherited and acquired anaemias differ by etiology and can affect required diagnostic tests and medications that can be prescribed during pregnancy.

G6PD deficiency, caused by mutations on the X-linked G6PD gene, is mainly asymptomatic unless affected individuals are exposed to certain medicines or foods that induce oxidative stress ( Cappellini & Fiorelli, 2008). This oxidative stress causes intravascular and extravascular haemolysis of G6PD deficient red blood cells with potentially serious clinical consequences. Heterozygous women, even with the same genotype, manifest a range of phenotypes from normal G6PD activity to severe deficiency, due to the early random inactivation of the X-chromosome (Lyonisation, ( Lyon, 1961)).

Haemoglobinopathies are caused by a number of mutations on the genes that encode haemoglobin alpha and non-alpha chains ( Weatherall, 2013). Alpha chains are encoded by four alpha genes located in pairs on the chromosome 16, while non-alpha chains are encoded by several genes on the beta-globin cluster on chromosome 11. Expression of non-alpha chains changes during embryonic, foetal and adult development; in children over one year of age and in adults, over 96% of haemoglobin is composed by two alpha and two beta chains(HbA) and between 2.2–3.5% by two alpha and two delta chains (HbA _2_). Mutations that cause imbalance among the four chains are called thalassaemias ( Harteveld & Higgs, 2010; Taher *et al.*, 2017). Hamoglobinopathies are associated with a spectrum of reduced haemoglobin levels, ranging from very mild (ca 0.5g/dL average reduction) to severe anaemia. The geographic distribution of abnormal haemoglobins corresponds to that of malaria before modern times, because these abnormalities confer some protection against malaria or its pathological effects ( Haldane, 1949; Hutagalung *et al.*, 1999; Siniscalco *et al.*, 1961; Taylor & Fairhurst, 2014; Weatherall *et al.*, 2010). The refugee and migrant populations living along the Thailand-Myanmar border, an area of low seasonal malaria transmission, are composed of several distinct ethnic groups: mainly Burman, Sgaw Karen and Poe Karen, followed by Mon, Kachin, Shan, and Rakhine. While a few studies have investigated the prevalence of haemoglobinopathies among Burman in central Myanmar ( Than *et al.*, 2005; Win *et al.*, 2005), little is known about Karen and other ethnic minorities living in Karen state and along the Thailand border. There are scattered reports from Karen and Burman patients who have been resettled in high-income countries where the capacity to determine detailed genetic traits is possible ( Lee, 2012; Phylipsen *et al.*, 2010). G6PD deficiency has been studied in recent years in this border population ( Bancone *et al.*, 2014; Phompradit *et al.*, 2011), but quantitative characterization of G6PD phenotypes in women has not been carried out yet at the population level.

The aim of this report is to describe the prevalence of IRD among the pregnant women attending routine antenatal screening at SMRU clinics along the Thailand-Myanmar border.

## Methods

### Study area and population

The Shoklo Malaria Research Unit is located in the north-western border of Thailand and has been providing free health assistance to the local refugee population for 30 years. The antenatal care (ANC) program was established for the early detection and treatment of malaria in 1986 for refugees and since 1998 for migrants. Women living in the catchment area of SMRU clinics are encouraged to attend the ANC as soon as they are aware of their pregnancy. Over 3,000 pregnant women of mainly Burman and Karen ethnicity register for ANC at SMRU clinics each year. SMRU’s free comprehensive antenatal, delivery and postpartum care has been described elsewhere ( Hoogenboom *et al*., 2015). At the first consultation, demographic information is collected, an obstetric and medical history recorded, and detailed clinical examination done. Antenatal screening from July 2012 to September 2016 included examination of IRB in line with Thailand national guidelines. All women had language appropriate group counselling at this first visit about the different screening tests and this was followed by one on one counselling with the option to opt out. Formal written consent was not required for the original routine blood sampling. Permission to waive consent from the individual patient for analysis of routine data was asked and obtained from the Ethic Committee of the Faculty of Tropical Medicine, Mahidol University (approval letter MUTM2017-041-01), and from Oxford University (OXTREC#583-16).

### Definition of ethnicity

Pregnant women were asked to report the ethnicity of both parents as belonging to one of the following groups based on locally preferred terms for self-identification: Sgaw Karen, Poe Karen, Burman, “Muslim”, Mon, Kachin, Pa Oh, Rakhine, Shan and “others”. The ethnicity of the woman was defined “mixed” when parents’ ethnicity differed. Of note, in this border area people of Islamic faith often self-identify as “Muslim” when asked about their ethnicity; the “Muslim” group is not an ethnic group, but rather a heterogeneous group of subjects with various ethnic backgrounds.

### Laboratory analyses

In the central Haematology Laboratory, G6PD deficiency was screened by the NADPH Fluorescent Spot Test (FST, R&D Diagnostic, Greece). The FST allows detection of G6PD activity in a small volume of blood by supplying the substrates for the G6PD-mediated reduction of NADP ^+^ to the naturally fluorescent NADPH. The fluorescence is detected in the mix spotted on paper by observation under long-wave UV light (ca 340nm) ( Beutler & Mitchell, 1968). Although the FST can show varying degrees of fluorescent intensity, samples are classified either as deficient or as normal based on visual assessment. The threshold of enzymatic activity which produces a visually normal fluorescence has been estimated to be approximately 30% of normal ( Bancone *et al.*, 2015). As a result, samples classified as deficient include most of the severely mutated homozygote and hemizygote subjects; samples classified as normal include specimens from wild type subjects and from subjects with intermediate activity over the 30% threshold (e.g heterozygous women or individuals with milder mutations). G6PD quantitative phenotype was assessed by spectrophotometry carried out at 37°C according to the standard WHO protocol on whole blood depleted of WBCs ( Beutler *et al.*, 1977) in a subsample of sequentially recruited women. G6PD activity was calculated after normalization with Hb concentration and expressed as IU/gHb. The G6PD activity population median of 11.5 IU/gHb was established previously in the laboratory using the same technique on G6PD normal males ( Bancone *et al.*, 2014). In the same selected women, G6PD genotyping was performed using established PCR-RFLP protocols ( Bancone *et al.*, 2014). Genotyping for Mahidol was performed on all these women, while Chinese-4, Kaiping, Canton and Mediterranean variants were analysed only on women with either enzymatic activity below 80% of normal in Mahidol-wild type genotype or enzymatic activity below 30% and Mahidol-heterozygous genotype. The minimum size of the subsample analysed for G6PD quantitative phenotyping and genotyping was set at 300 subjects; this was calculated to allow at least 5 subjects in the smaller genotype group (homozygous mutated) based on the expected allele frequency of 15%.

### Haemoglobinopathies

For haemoglobin typing, blood samples were analysed either by high-performance liquid chromatography (HPLC, using Biorad D-10™) at the local Mae Sot hospital or by Capillary Electrophoresis (CE, using a Capillarys II, Sebia, France) at the central SMRU Haematology Laboratory. Adult haemoglobin contains mainly HbA (2α2β, 96–98%) and HbA _2_ (2α2δ, 2.2–3.5%). Both HPLC and CE allow for diagnosis of beta-thalassaemia carriage (by mean of increased percentage of HbA _2_, usually over 3.5%), presumptive diagnosis of alpha-thalassaemia carriage (by mean of decreased percentage of HbA _2_, usually under 2.2%) and diagnosis of Hb structural variants such as HbE, HbC, HbS (by mean of appearance of retention peaks at specific elution times). Haemoglobin Constant Spring (HbCS), a non deletional alpha thalassaemia caused by a TAA>CAA mutation on the termination codon of alpha gene, was diagnosed by CE only, with a peak ≥0.5% in the HbC/SC retention zone. Common Asian deletional mutations on the alpha globin genes (3.7, 4.2, SEA) were analysed using a modified multiplex gap-PCR protocol ( Chong *et al*., 2000). Molecular analysis was performed on an arbitrarily minimum sample size of 200 subjects with presumptive diagnosis of alpha-thalassaemia defined by low percentage of HbA _2_ (≤2.2% by HPLC or ≤2.1% by CE) in order to assess correlation between genotype and percentage of HbA _2_; an additional sample of at least 150 subjects with beta-thalassaemia trait was studied in order to assess prevalence of alpha-thalassaemia in this group and analyse variation of percentage of HbA _2_ in subjects with mutations on both alpha and non-alpha globin chains.

### Statistical analysis

All women with available data in the timeframe of the study were included in the analysis. Data were entered and analysed in SPSS IBM SPSS Statistics for Windows, version 23.0 (IBM Corp., Armonk, N.Y., USA). Chi-square test was used for comparison of allelic frequencies among different ethnic groups. Normality of G6PD activity was tested by the Kolmogorov-Smirnov test.

## Results

**Figure 1.**
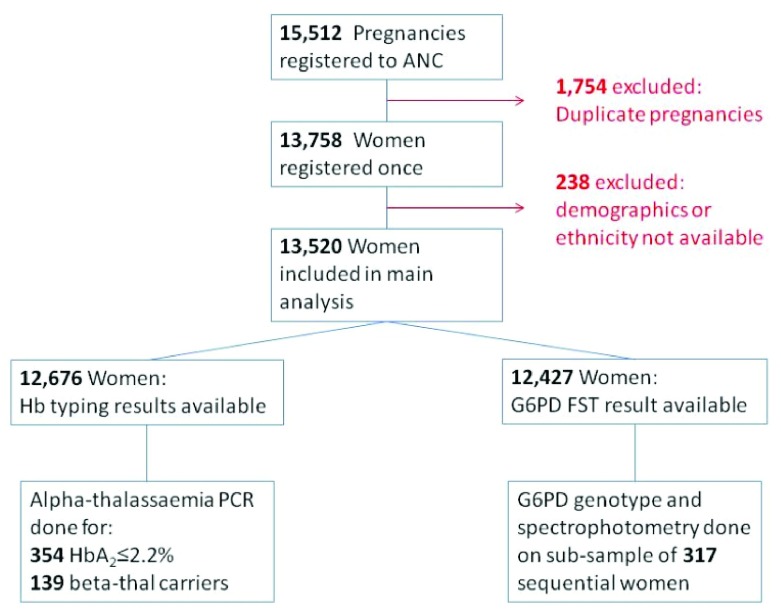
Study flowchart.

Study flow is summarized in Figure 1. A total of 15,512 pregnancies were registered at the ANC between July 2012 and September 2016. For women with more than one pregnancy during this time frame, successive pregnancies (n=1,754) were excluded from analysis. Complete demographic and ethnicity information were missing for 238 women, leaving a total of 13,520 analyzable women. G6PD FST screening and haemoglobin typing results were available in over 90% of women. Additional data on G6PD genotype and quantitative phenotype were analysed in a subsample of 317 sequential women who attended the clinics between August and September 2012. Between August 2012 and August 2013, alpha deletional mutations were analysed by PCR on 354 women presenting with a low percentage of HbA _2_ (≤2.2% by HPLC or ≤2.1% by CE) and on 159 women with beta-thalassaemia trait.

### Prevalence and distribution of ethnic groups

A total of 12,605/13,520 (93.2%) women reported both parents to be of the same ethnicity and were therefore assigned to one of the nine common ethnic groups or to the pooled category of uncommon ethnic groups called “others” ( Table 1). Overall, the most represented ethnic group was the Sgaw Karen (44.3%), followed by Burman (28.5%), Poe Karen (12.4%) and “Muslim” (4.4%). The distribution of these four major ethnic groups reflected those of the general population attending the SMRU clinics with the Sgaw Karen comprising the vast majority of women attending in the refugee camp of MLA (71.3%) and Burman women representing 39.7% and 47.6% of the population attending the clinics for migrant population of WPA and MKT, respectively. Poe Karen were 9.1% to 16.9% in the three clinics, while “Muslim” were mainly in MLA camp for displaced persons (11.9%). The other ethnic groups were in very low numbers in all clinics.

**Table 1.** Distribution of ethnic groups in the three Shoklo Malaria Research Unit clinics.

Ethnicity	MKT		WPA		MLA		Total	
Sgaw Karen	1,081	26.3%	1,470	32.0%	3,443	71.3%	5,994	44.3%
Poe Karen	456	11.1%	774	16.9%	441	9.1%	1,671	12.4%
Burman	1,954	47.6%	1,822	39.7%	77	1.6%	3,853	28.5%
“Muslim”	15	0.4%	11	0.2%	573	11.9%	599	4.4%
Mon	72	1.8%	56	1.2%	14	0.3%	142	1.1%
Kachin	2	0.0%	0	0.0%	12	0.2%	14	0.1%
Pa Oh	46	1.1%	501	1.1%	10	0.2%	106	0.8%
Rakhine	55	1.3%	14	0.3%	1	0.0%	70	0.5%
Shan	9	0.2%	10	0.2%	6	0.1%	25	0.2%
“Mixed”	348	8.5%	334	7.3%	234	4.8%	916	6.8%
Other	66	1.6%	48	1.0%	16	0.3%	130	1.0%
**Total**	**4,104**	**100.0%**	**4,585**	**100.0%**	**4,827**	**100.0%**	**13,520**	**100.0%**

### G6PD phenotype by fluorescent spot test

A total of 12,427/13,520 (91.9%) women were screened for G6PD activity showing an overall prevalence of phenotypic deficiency of 2.9% ( Table 2). Sgaw Karen and Mon ethnicities showed the highest prevalence of deficiency (over 4%), while all the other ethnic groups had less than 2% prevalence, with the “Muslim” and Rakhine at the lower end (close to 1%).

**Table 2.** G6PD phenotype by fluorescent spot test according to ethnic group.

	Deficient	Normal	Total	% phenotypic deficiency
Sgaw Karen	226	5,110	5,336	4.2%
Poe Karen	25	1,525	1,550	1.6%
Burman	72	3,618	3,690	2.0%
“Muslim”	6	504	510	1.2%
Mon	7	128	135	5.2%
Kachin	0	12	12	0.0%
Pa Oh	1	99	100	1.0%
Rakhine	0	69	69	0.0%
Shan	1	23	24	4.2%
“Mixed”	20	860	880	2.3%
Other	2	119	121	1.7%
**Total**	**360**	**12,067**	**12,427**	**2.9%**

### G6PD genotypes according to ethnic group

Among the subsample of 317 women, G6PD genotyping was performed for Mahidol, Canton, Kaiping, Chinese-4 and Mediterranean variants. Since the minor ethnic groups were hardly represented in this smaller sample, they were pooled together. The major mutation found was Mahidol, representing over 95% of all mutations in all ethnic groups, with the exception of Burmans in whom Kaiping, Canton and Chinese-4 were found globally at a polymorphic frequency ( Table 3).

**Table 3.** G6PD genotypes among 317 women of different ethnicities.

	Wild type	Mahidol heterozygote	Chinese-4 heterozygote	Kaiping heterozygote	Mahidol homozygote	Mahidol- Canton	Total	Overall allelic frequency of all variants	Allelic frequency of Mahidol variant
Sgaw Karen	91	47	1	1	7	0	147	21.4%	20.7%
Poe Karen	34	12	0	1	4	0	51	20.6%	19.6%
Burman	67	9	1	2	0	1	80	8.8%	6.3%
“Muslim”	11	0	0	0	0	0	11	0.0%	0.0%
Other	6	1	0	0	0	0	7	7.1%	7.1%
“Mixed”	16	5	0	0	0	0	21	11.9%	11.9%
**Total**	**225**	**74**	**2**	**4**	**11**	**1**	**317**	**16.3%**	**15.3%**

### Quantitation of G6PD activity by spectrophotometry

**Figure 2.**
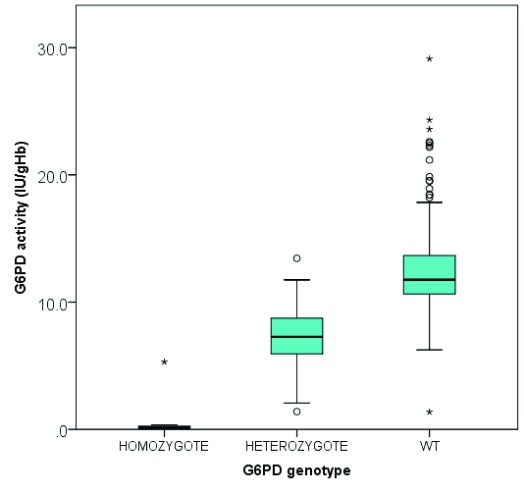
G6PD activity (IU/gHb) assessed by spectrophotometer in 317 women.

**Figure 3.**
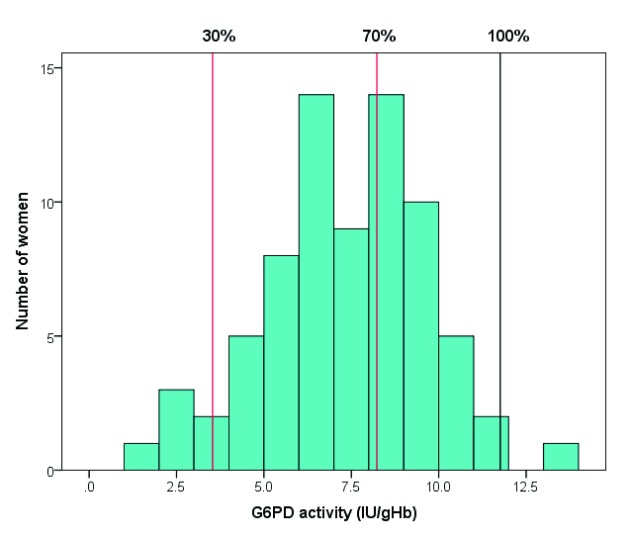
Distribution of G6PD enzymatic activities among 74 Mahidol heterozygous women.

The distribution of quantitative G6PD activity in 317 women is displayed in Figure 2, according to G6PD genotype (all mutations are pooled). G6PD activity in homozygous women for wild type allele was not normally distributed and had a median (IQR) of 11.76 (10.61-13.64) IU/gHb, similar to the median of 11.50 IU/gHb assessed previously in males; G6PD activity in the deficient homozygotes was not normally distributed and had a median (IQR) of 0.13 (0.11-0.25) IU/gHb. Both homozygous genotypes were therefore at the extremes of the activity spectrum, while heterozygous women had a wide distribution of activities from completely normal to “completely” deficient. The G6PD activity in 74 heterozygous women for the most prevalent Mahidol variant ( Figure 3) showed a normal distribution with a mean (SD) of 7.38 (2.33) IU/gHb corresponding to 62.8% of the normal activity (based on the population median activity in females). According to this distribution, 6.6% of women had a G6PD activity in the range of deficiency (<30% normal activity), 60.5% were in the 30–70% activity range, and the remaining 32.9% were in the normal activity range (>70% normal activity).

Vertical red lines are the 30% and 70% of normal activity (based on the female population median, 100%).

### Haemoglobin variants

Results of the haemoglobin typing by HPLC or Capillary Electrophoresis among 12,676 women are shown in Table 4. The highest HbE allelic frequency was found among women of Rakhine (23.2%) and Burman (11.0%) followed by the Mon ethnicities (7.7%). Sgaw Karen and Poe Karen had the lowest HbE allelic frequency among all the ethnic groups (1.0% and 1.7% respectively). Beta-thalassaemia had an allelic frequency lower than 5% in all ethnic groups, with a carrier prevalence less than 10%; the highest allelic frequency was found in Sgaw Karen and Poe Karen (3.9%) and the lowest among women of the Rakhine and Burman ethnicities (0.9% and 0.7%, respectively). Haemoglobin Constant Spring (diagnosed only by capillary electrophoresis) was found in less than 2% of the women, with a higher prevalence among Burman (1.9%) and the lowest among Sgaw Karen (0.7%).

**Table 4.** Distribution of Hb variants diagnosed by HPLC or CE in the different ethnic groups.

	Sgaw Karen	Poe Karen	Burman	“Muslim”	Mon	Pa On	Rakhine	Shan	Kachin	Other	“Mixed”	Total
Hb Normal ^&^	4,907	1,388	2,815	473	110	87	39	20	10	102	752	10,703
Beta-thalassaemia trait	421	123	60	17	4	4	0	1	1	3	49	683
Beta-thalassaemia trait with HbCS	3	1	0	0	0	0	0	0	0	0	0	4
Beta-thalassaemia/ HbE disease	1	1	3	1	0	0	1	1	0	0	1	9
HbEE	0	1	39	0	1	2	4	0	0	0	2	49
HbE trait	103	51	726	30	19	9	24	2	1	20	71	1,056
HbE trait with HbCS	2	1	14	0	0	0	0	0	0	0	0	17
HbCS	34	17	58	7	2	2	0	0	1	1	8	130
Suspected HbH disease (HbA _2_≤1.0)	2	2	6	0	0	0	0	0	0	0	0	10
Unknown Abnormal variant	6	1	6	0	0	0	1	0	0	0	1	15
Total	5,479	1,586	3,727	528	136	104	69	24	13	126	884	12,676
HbE carrier prevalence	1.9%	3.4%	21.0%	5.9%	14.7%	10.6%	42.0%	12.5%	7.7%	15.9%	8.4%	8.9%
HbE allelic frequency	1.0%	1.7%	11.0%	2.8%	7.7%	6.3%	23.2%	4.2%	3.8%	7.9%	4.2%	4.6%
Beta-thalassaemia carrier prevalence	7.8%	7.9%	1.7%	3.4%	2.9%	3.8%	1.4%	8.3%	7.7%	2.4%	5.7%	5.5%
Beta- thalassaemia allelic frequency	3.9%	3.9%	0.8%	1.7%	1.5%	1.9%	0.7%	4.2%	3.8%	1.2%	2.8%	2.7%
HbCS carrier frequency	0.7%	1.2%	1.9%	1.3%	1.5%	1.9%	0.0%	0.0%	7.7%	0.8%	0.9%	1.2%

^&^ This includes all subjects with HbA _2_>1.0 HbCS= Haemoglobin Constant Spring

### Mutations on the alpha globin chains

Among the 354 subjects with low HbA _2_ and characterised by PCR ( Table 5) less than half (45.2%) had mutations in the alpha genes, indicating that the percentage of HbA _2_ alone was not a good indicator of alpha-thalassaemia carriage. The lowest HbA _2_ percentage was associated with deletions on three alpha genes (HbH disease), (P<0.01 *vs* all others), while subjects with two or one mutation had increasingly higher HbA _2_ percentage, but not significantly different from those without mutations ( Table 5). Since this was a selected group of women, the prevalence and distribution of mutations analysed (3.7, 4.2 and SEA) cannot be considered representative of the general pregnant population. Among the consecutively enrolled 159 beta-thalassaemia carriers the prevalence of concurrent alpha-thalassaemia carriage was 25.3% and in this group there was no difference in percentage of HbA _2_ between subjects with alpha deletions and those without. Overall, the most common deletion was -3.7 followed by SEA.

**Table 5.** Median HbA _2_ percentage according to alpha chain genotype in subjects with presumptive alpha-thalassaemia or beta-thalassaemia trait.

Genotype	Presumptive alpha-thalassaemia (HbA _2_≤2.2)	Median (IQR) %HbA _2_	Beta-thalassaemia trait	Median (IQR) %HbA _2_
Normal	194	1.90 (1.80-2.10)	119	5.20 (4.70-5.70)
αα/α-3.7	124	1.90 (1.80-2.00)	37	5.20 (4.60-5.80)
αα/α -4.2	1	1.70		
αα/-SEA	8	1.75 (1.63-2.12)		
α-3.7/α-3.7	20	1.85 (1.80-1.90)	3	6.60
α-3.7/-SEA (HbH)	7	0.60 (0.40-1.00)		

## Discussion

In Myanmar, the population is divided officially into eight main ethnicities, which the government has further classified into 135 different indigenous ethnic groups. The majority group Burman make up 68% of the country’s population of 55 million, with the Shan (9%), Karen (7%), Rakhine (or Arakanese) (4%) and Mon (2%) comprising the largest minority ethnic groups. In this area of the Thailand-Myanmar border, the Karen group predominates and this is further classified into Sgaw and Poe. Patients of Sgaw Karen, Burman, Poe Karen and “Muslim” ethnicity represent the four local major ethnic groups who seek health care at border SMRU clinics.

G6PD deficiency is relatively common in all ethnic groups with phenotypic deficiency prevalence in women ranging from 1% of most groups (corresponding to the observed allelic frequency of 10%) to a maximum of over 4% (corresponding to the observed allelic frequency of 20%) in the Sgaw Karen. These data confirm previous observations in the male population on the Thailand-Myanmar border ( Bancone *et al.*, 2014; Louicharoen *et al.*, 2009; Phompradit *et al.*, 2011), inside Myanmar ( Arnolda *et al.*, 2015; Matsuoka *et al.*, 2004; Nuchprayoon *et al.*, 2008) and in Kachin state adjacent to the Myanmar-China border ( Li *et al.*, 2015). Current G6PD qualitative field tests can only detect marked deficiency, and women classified as “G6PD normal” are a rather heterogeneous group of subjects with G6PD activity ranging from 30% to 100% of normal, whose haemolytic risk when treated with oxidative drugs varies widely. Characterization of quantitative G6PD phenotype in a large population of females has never been carried out before and represents a resource for informing treatment for several infectious diseases in women living in an endemic area. Nitrofurantoin, a known precipitant of haemolysis in G6PD deficient patients ( Youngster *et al.*, 2010), is recommended as first line treatment for urinary tract infections in pregnancy ( Gupta *et al.*, 2011), due to its safety, low cost, and efficacy. Primaquine cannot be prescribed in pregnant women, but it is indicated postpartum for women infected with *P. vivax* during pregnancy and is a cornerstone of vivax malaria elimination. Our data show that over 60% of heterozygote women have a G6PD enzymatic activity over the threshold of deficiency for field tests but in the intermediate range (i.e. lower than the normal), and so could be at risk of drug induced haemolysis with certain primaquine regimens ( Chu *et al.*, 2017). Furthermore, the slightly skewed distribution of G6PD activities among heterozygous women might suggest some somatic selection against deficient RBCs as seen in the severe variant Mediterranean ( Rinaldi *et al.*, 1976); more investigations will be needed to establish whether this is specific to pregnancy or to the Mahidol variant.

Haemoglobin variants are common in this population, affecting at least 15% (HbE and beta-thalassaemia) and possibly up to more than 40% (including alpha-thalassaemia) of women overall. There are marked differences in the prevalence of HbE and beta-thalassaemia in different ethnic groups (as described previously ( Win *et al.*, 2005)) and this peculiar distribution, combined with a low rate of intermarriage between ethnic groups, suggests that the population may have partial protection from the deleterious beta-thalassaemia/HbE syndrome. In fact only 9 women in the entire cohort (0.07%) were found to have beta-thalassaemia/HbE syndrome, significantly below what would be expected in a random mating population (χ ^2^=23.1, P<0.001). Furthermore, within the Sgaw Karen ethnicity only, the observed frequency of beta-thalassaemia/HbE genotype was lower than expected (χ ^2^=9.14, P<0.05) suggesting a further associated reduced survival to reproductive age. Molecular characterization of alpha-thalassaemia was only performed in subjects with a presumptive diagnosis by HPLC or CE and in a subsample of beta-thalassaemia carriers. Data presented here confirm that, with the exclusion of HbH disease, a percentage of HbA _2_ ≤2.2 was not specific for alpha-thalassaemia trait, as over half of the women identified by this criteria had a normal genotype ( Van Delft *et al.*, 2009). The assessed higher prevalence of alpha-thalassaemia among the beta-thalassaemia carriers (25.3%) seems to be closer to the true prevalence in the population and it is higher than that estimated only by the low percentage of HbA _2_ (16.9%).

While the main reasons for investigating G6PD deficiency and Hb variants in pregnant women is the clinical management of anaemia and treatment with antimicrobial agents, the results carry implications for the offspring of the women tested. Communicating this type of information to women in this population with low health literacy in meaningful language is challenging. When G6PD deficiency is diagnosed, the staff counsel the woman about which drugs and food should be avoided and give her a card explaining the diagnosis and contraindicated medications. When beta-thalassaemia and HbE are diagnosed, the woman is informed that she might experience weakness or other anaemia symptoms during the pregnancy and a card with the diagnosis and a short description is given to the patient. During this simple counselling the woman is informed she might have passed her genetic trait to her present foetus and might pass it in future pregnancies; the possibility that the offspring might inherit the abnormal Hb trait from both parents and be severely affected represents an important, but difficult to convey, part of the counselling about haemoglobinopathies. Due to fragmented education affected by conflict, poverty and migration, approximately 50% of ANC attendants are illiterate ( Carrara *et al.*, 2011), and the majority are totally unfamiliar with concepts of genetics and inheritance. The challenge of counselling about often asymptomatic diseases with complex implications is significant ( McGready *et al.*, 2014) and could result in dire unintended consequences in some individuals. In countries where haemoglobinopathies are common, several screening approaches have been developed (for a review see Amato *et al.*, 2014). Cost/benefit studies have shown that prevention programs are highly cost effective ( Koren *et al.*, 2014) and a means to reduce suffering for patients and families ( Ballantyne *et al.*, 2009). In Myanmar, there is no routine practice of prenatal screening for Hb variants, and this kind of testing would only be available to private patients consulting at specialty clinics in major urban centres. In Thailand, the screening is performed by HPLC on voluntary basis at the first ANC appointment. When the mother is found to be a carrier, the father of the baby is also tested and if found to be carrier, molecular investigations are performed. In the low-resource setting of SMRU, Hb testing for the mother alone costs approximately the same amount as all other investigations performed at the first ANC consultation combined. Hb testing for the father is not routinely performed due to cost, and pre-natal foetal diagnosis is not possible. Furthermore, SMRU is not equipped to offer long-term clinical care for subjects with transfusion-dependent thalassaemia, and the costs for refugees or migrants would be a limiting factor even when referral to a reference centre in Thailand is possible. Safe termination of pregnancy is not accessible to most migrant and refugee women in this border area and unsafe abortions are a cause of maternal morbidity and mortality in these vulnerable communities ( Turner *et al.*, 2012). Despite these challenges and limitations, the current data on the high prevalence of HbE, alpha and beta-thalassaemia warrant a continuation of screening and encourage the implementation of a more extended program of early information and counselling to girls and women of reproductive age among the population. The pioneering and remarkable example of the thalassaemia screening carried out by the “Centro Studi Microcitemie Roma” during 37 years among young students in Latium, Italy, ( Amato *et al.*, 2014) shows that information, screening of relatives, and counselling are major factors in the success of programs for the prevention of severe haemoglobinopathies. For long term sustainability, laboratory testing can be carried out with cheaper techniques, such as single-tube osmotic fragility test (OFT), dichlorophenolindophenol (DCIP) for HbE, and microscopic examination following a step-wise approach whereby the most expensive tests are only performed on samples that result abnormal by initial screening. This approach, especially if offered as part of a strong preconception healthcare package, including partner testing where appropriate, genetic counselling, folic acid supplementation, and effective and acceptable family planning provision, could substantially decrease the suffering of vulnerable families. Such a program would require community engagement and human resource development to equip local staff with the specialized skills of sensitive and responsive genetic counselling for individuals with limited background science knowledge.

In conclusion, the current data show that G6PD deficiency and abnormal haemoglobins are common among pregnant women in this area of the Thailand-Myanmar border, and likely contribute to increase the proportion of women with anaemia. In women with congenital Hb variants, anaemia can be present before pregnancy and might worsen during gestation, and requirement for iron supplementation can be difficult to assess. The current population data will help inform ongoing efforts to optimize the clinical management of anaemia in the local pregnant population by investigating newer marker of iron deficiency anaemia (for example, hepcidin), which might be more reliable in such context as compared to classic markers ( Bah *et al.*, 2017; Wray *et al.*, 2017).

## Data availability

The data referenced by this article are under copyright with the following copyright statement: Copyright: © 2017 Bancone G et al.

Due to ethical and security considerations, the data that supports the findings in this study can be accessed only through the Data Access Committee at Mahidol Oxford Tropical Medicine Research Unit (MORU). The data sharing policy can be found here: http://www.tropmedres.ac/data-sharing. The application form for datasets under the custodianship of MORU Tropical Network can be found in Supplementary File 1.

## Ethical statement

Ethical approval for the study was granted by the Faculty of Tropical Medicine, Mahidol University (MUTM 2017-041-01) and by Oxford University (OXTREC#583-16).

## Supplementary material

Supplementary File 1: Application form for datasets under the custodianship of Mahidol Oxford Tropical Medicine Research Unit (MORU) Tropical Network.
