# Supplementary material for: Prevalences of inherited red blood cell disorders in pregnant women of different ethnicities living along the Thailand-Myanmar border
Source: Wellcome Open Res. 2017 Nov 2;2:72. Originally published 2017 Aug 24. [Version 2] doi: 10.12688/wellcomeopenres.12338.2 (PMC5686509; doi:10.12688/wellcomeopenres.12338.2)
Supplement: Peer review history

# Peer review: Referee response for version 2

**DOI:** 10.21956/wellcomeopenres.14103.r27543

**Published:** 2017-11-13

**Authors:** Issarang Nuchprayoon

**Affiliations:** 1 Faculty of Medicine, Chulalongkorn University, Bangkok, Thailand

I have read the revised version and all points were addressed.  The responses and reasons were reasonable. Well done!

I have read this submission. I believe that I have an appropriate level of expertise to confirm that it is of an acceptable scientific standard.

# Peer review: Referee response for version 2

**DOI:** 10.21956/wellcomeopenres.14103.r27542

**Published:** 2017-11-02

**Authors:** Thomas N. Williams

**Affiliations:** 1 Centre for Geographic Medicine Research-Coast, Kenyan Medical Research Institute (KEMRI)-Wellcome Trust Research Programme, Kilifi, Kenya; 2 Imperial College, London, UK

Thanks for these changes. I think the manuscript is now acceptable. Well done!

I have read this submission. I believe that I have an appropriate level of expertise to confirm that it is of an acceptable scientific standard.

# Peer review: Referee response for version 1

**DOI:** 10.21956/wellcomeopenres.13359.r25455

**Published:** 2017-09-25

**Authors:** Issarang Nuchprayoon

**Affiliations:** 1 Faculty of Medicine, Chulalongkorn University, Bangkok, Thailand

This study is a valuable study based on its large sample size of pregnant women of rarely studied ethnic group, namely Sgaw Karen in comparison with Burman. 

 The subject of study; inherited red cell variants are valid in itself. It is controversial whether heterozygous G6PD mutation, thalassemia traits, and ABO, Rh blood groups are 'disorders'. People with all of these conditions do not have anemia in their usual state. Only people with compound heterozygous thalassemia 'diseases', homozygous HbE (HbEE), and HbH will have some degree of anemia. The causes of anemia in pregnant women are by far commonly caused by iron deficiency, which is not addressed in this study. Therefore the reference to anemia in pregnancy should be de-emphaized in the rationale and conclusion of this study. 

 G6PD results in this report does confirm other studies in Myanmar ethnic groups, many of which were reported in males. One additional citation that the author should consider is a report from Louicharen *et al*, 2009 ^1^, who studied prevalence of G6PD mutations in Karen of suan peung, at a Thai-Myanmar border. In this paper in which details are reported in supplementary article in Science website. the prevalence of G6PD deficiency 20%, with predominantly (94%) Mahidol variant. 

 The methodology for estimating prevalence of alpha-thalassemia trait in this paper is controversial. Using  low HbA2 is known to be unreliable and in fact the authors showed only 44.1% specificity. Since it is known how much is alpha-tha are missed in people with normal A2, the total prevalence is therefore not known for certainty.  The prevalence of alpha-thalassemia in subset of studies in known beta-thal trait population may be more accurate estimation assuming all subjects were tested, but need to be stratified by each ethnic group. The author may wish state a conservative estimate in each ethnic group, like at least 25%, instead of a specific proportion.

 Due to large number of Karen and Burmans in this study, the author may wish to point out the value of this paper in population genetics. Their difference in prevalence was a founder effect and due to estimation of G6PD Mahidol variant emerging around 1500 years ago ^1^ as protective factor against malaria, the Burman may be a more recent population in this region. 

 In the final conclusion paragraph, the author should made a summary statement of their key findings on prevalence of variants but not about anemia since this paper did not address the issue. A mention of newer marker for iron deficiency detection is irrelevant and not useful in this resource-limited region. Suggestion of counseling is irrelevant and impractical here.

I have read this submission. I believe that I have an appropriate level of expertise to confirm that it is of an acceptable scientific standard, however I have significant reservations, as outlined above.

## References

[1] LouicharoenCPatinEPaulRNuchprayoonIWitoonpanichBPeerapittayamongkolCCasademontISuraTLairdNMSinghasivanonPQuintana-MurciLSakuntabhaiA: Positively selected G6PD-Mahidol mutation reduces Plasmodium vivax density in Southeast Asians. *Science*.2009;326(5959) : 10.1126/science.1178849 1546-9 10.1126/science.1178849 20007901

[2] GalanelloRCaoA: Gene test review. Alpha-thalassemia. *Genet Med*.2011;13(2) : 10.1097/GIM.0b013e3181fcb468 83-8 10.1097/GIM.0b013e3181fcb468 21381239

# Response

**Published:** 2017-10-23

**Authors:** Germana Bancone

**Affiliations:** Shoklo Malaria Research Unit, Thailand

We thank the reviewer for his useful comments and suggestions. Below the answer to his questions.

 Q1) The subject of study; inherited red cell variants are valid in itself. It is controversial whether heterozygous G6PD mutation, thalassemia traits, and ABO, Rh blood groups are 'disorders'. People with all of these conditions do not have anemia in their usual state. Only people with compound heterozygous thalassemia 'diseases', homozygous HbE (HbEE), and HbH will have some degree of anemia. The causes of anemia in pregnant women are by far commonly caused by iron deficiency, which is not addressed in this study. Therefore the reference to anemia in pregnancy should be de-emphaized in the rationale and conclusion of this study. 

 A1) Anaemia is the most common medical problem of the pregnant population under study and is associated with  maternal mortality ( McGready, Boel et al. 2012) but a systematic analysis of genetic causes of anemia has never been undertaken. We think that, although analyzing these markers is interesting in itself, we cannot avoid highlighting that our primary interest is their contribution to anaemia during pregnancy; our plan is also to analyse soon clinical data in relation to the findings presented in this manuscript. Following the reviewers suggestions, we have re-phrased some of the text in the introduction and conclusion to clarify our message.

 Q2) G6PD results in this report does confirm other studies in Myanmar ethnic groups, many of which were reported in males. One additional citation that the author should consider is a report from Louicharen *et al*, 2009 ^1^, who studied prevalence of G6PD mutations in Karen of suan peung, at a Thai-Myanmar border. In this paper in which details are reported in supplementary article in Science website. the prevalence of G6PD deficiency 20%, with predominantly (94%) Mahidol variant. 

 A2) We have added the suggested citation

 Q3) The methodology for estimating prevalence of alpha-thalassemia trait in this paper is controversial. Using low HbA2 is known to be unreliable and in fact the authors showed only 44.1% specificity. Since it is known how much is alpha-tha are missed in people with normal A2, the total prevalence is therefore not known for certainty.  The prevalence of alpha-thalassemia in subset of studies in known beta-thal trait population may be more accurate estimation assuming all subjects were tested, but need to be stratified by each ethnic group. The author may wish state a conservative estimate in each ethnic group, like at least 25%, instead of a specific proportion.

 A3) As we answered also to the other reviewer, we have modified all the text and Tables to improve accuracy and clarity on this point.

 Q4) Due to large number of Karen and Burmans in this study, the author may wish to point out the value of this paper in population genetics. Their difference in prevalence was a founder effect and due to estimation of G6PD Mahidol variant emerging around 1500 years ago ^1^ as protective factor against malaria, the Burman may be a more recent population in this region. 

 A4) Although this is an interesting point to consider, the current data were not collected or analyzed to support or contradict evolutionary dynamics of Karen and Burman populations, therefore we prefer not to address the topic.

 Q5) In the final conclusion paragraph, the author should made a summary statement of their key findings on prevalence of variants but not about anemia since this paper did not address the issue. A mention of newer marker for iron deficiency detection is irrelevant and not useful in this resource-limited region. Suggestion of counseling is irrelevant and impractical here. 

 A5) We have revised the text of the conclusive paragraph. We strongly believe that, having found a pretty high prevalence of abnormal Hb variants, a discussion on counselling pregnant women is warranted in this setting. Though this counselling is time-intensive and challenging, it is feasible in low resource settings with appropriately trained and sensitive local staff.

 Furthermore, better diagnostic tools for iron-deficient anaemia during pregnancy have been shown to be particularly useful in populations with a high prevalence of haemoglobin disorders ( Wray, Allen et al. 2017); our data bring evidence on the widespread distribution of IRD and on the need for testing in resource-limited areas.

# Peer review: Referee response for version 1

**DOI:** 10.21956/wellcomeopenres.13359.r25454

**Published:** 2017-09-04

**Authors:** Thomas N. Williams

**Affiliations:** 1 Centre for Geographic Medicine Research-Coast, Kenyan Medical Research Institute (KEMRI)-Wellcome Trust Research Programme, Kilifi, Kenya; 2 Imperial College, London, UK

This is a nice summary of an impressive study of red cell disorders in more than 13,000 pregnant women by the team at the Shoklo Malaria Research Unit on the Thai-Myanmar border.

**Major comments**

 1) The background to the study is rationalized on the basis of the potential importance of inherited red cell disorders as causes and confounders of anaemia in pregnant women within the region.  The inclusion of data on ABO and Rhesus in this paper, though interesting, does not fit well within this rational framework.  Neither are particularly relevant to the main story. I would either drop those data or alter the rationale to justify their inclusion. 

 2) The referencing could be improved throughout the introduction. For example, a reference for Lyonisation and G6PD activity could be added, and the third paragraph, which contains a lot of technical information, should be referenced along the way rather than through the bunching of 4 (slightly random) references at the end.

 3) Introduction paragraph 3 could be tightened up. The first sentence is a bit confused: “..structural variants and deletional mutations on the hemoglobin …. chains” is a bit of a mixture of nomenclature more usually specific to either the protein or the gene.  The “deletional mutations” more commonly refer to the gene than the protein and probably more correctly should be referred to as “genetic deletions” rather than mutations (which more commonly relate to sequence changes than deletions).  Further down, “…97% of hemoglobin is composed of two alpha and two beta chains” – the authors should clarify that they’re talking about ADULT haemoglobin (HbA). The references on the geographic distribution with malaria are a bit random and refer to different IRDs. A recent authoritative review would probably be a better way to go.  

 4) Methods: Lab analyses. It would be very helpful if the authors explained here on behalf of the non-expert what the G6PD spot test is designed to do and at what level of normal G6PD activity the test turns positive. A major issue with this test, as the authors acknowledge, is that it is a binary test that shows sufficient / deficient only. It is not designed to pick up heterozygotes and is only partially sensitive for homozygotes, depending on the genotype involved and the degree to which it perturbs G6PD production.  Some discussion of this in the methods section would help the non-expert reader.

 5)  In the same section, the authors state that the quantitative test was conducted in “selected women”. It took me some digging to find that it was actually conducted in “a sub-set of sequentially recruited [and therefore “unselected”] women”. This could be better phrased at this point.

 6) It would help the reader if the flow chart (Figure 1) could be amended to make the above more clear with regard to the quantitative test and the genotyping for both G6PD and other conditions. 

 7)  Methods; haemobloginopathies.  The authors used an HbA2 of <2.2 or <2.1% (depending on the method) to define “presumptive” carrier status for alpha-thalassaemia.  As the authors state later in the paper, this is neither a sensitive nor specific approach to the diagnosis of the alpha-thalassaemias overall. It’s almost useless for the carrier forms of the common minor single gene -3.7 and -4.2kb deletional forms of the condition.  As such, the authors’ focus on this sub-group for further work up for alpha thalassaemias constitutes a biased sample.  It would really have been better from the perspective of trying to determine the prevalence of these conditions if the authors had centred their genetic testing on an unselected sample of women.  This is the approach they took for G6PD genotyping which therefore produced figures that were more understandable. The figures given for the prevalence of the various alpha thalassaemias are difficult to interpret and probably very misleading because of the way in which the patients were sampled.

 8) For this reason, I would remove the rows regarding “suspected alpha thalasssaemia” from table 5 – the authors say later on that half of the sub-sample they tested were normal genetically, so this row will mislead the casual reader. HbCS and HbE are more legitimate as these have their own HPLC peaks.

 9) Discussion, paragraph 2 seems a bit odd and comes rather out of the blue. This is the first mention of anthropology that I can find in the paper and the use of these IRDs as anthropological tools is put up and shot down in a single sentence!

 10) Authors could be more clear that their estimate of G6PDd on the basis of their spot test result will be an underestimate, since homozygosity for less severe variants will not always be picked up (as shown by the outlier on Fig 2).

 11) Discussion page 10 paragraph 2 left column. The authors say that all women with “Hb variants” are informed of the results and counseled that they may experience weakness etc in pregnancy.  I assume that this is not the case for “suspected alpha thalassaemia”? This would give rise to a great deal of unnecessary anxiety if half don’t actually have the condition anyway and in the case of the -3.7 and -4.2 deletions they are clinically silent when inherited alone.  The whole area of how to approach antenatal counseling in populations with a high prevalence of multiple conditions is highly complex and the discussion of this element could be tightened up a bit.  

**Minor**

 1) Though it’s an editorial decision, I would have assumed that since this is a UK based journal the spelling should be in UK English throughout.

 2) Abstract Methods: Blood groups were “diagnosed” makes it sound as if these are a disease! I would change to “determined” or similar.

 3) “resource rich countries” – preferred current term is “high-income countries”

 4) Final paragraph of the introduction could probably be condensed and moved to join the methods section. I would replace by a short sentence summarizing the aims of the current study.

 5) Please give the make and model of the HPLC machine used for the haemoglobin phenotyping.

 Could do with a good proof-read throughout. A few exapmples I picked up:

 1) Introduction line 3 “…living in area of past…” should read “…living in areas of past…”

 2) Abstract Methods: Data were available rather than data was available (data are plural in this context).

 3) page 4 line one column 2 – “SAE” should read “SEA”.

 4) Table 6 – use the Greek symbol for alpha rather than the letter A.

 5) page paragraph 2 left column “found to be carrier..” should read “found to be carriers..”

I have read this submission. I believe that I have an appropriate level of expertise to confirm that it is of an acceptable scientific standard, however I have significant reservations, as outlined above.

## References

[1] BanconeGGilderMChowwiwatNGornsawunGWinEChoWMooEMinACharunwatthanaPCarraraVWhiteNNostenFMcGreadyR: Prevalences of inherited red blood cell disorders in pregnant women of different ethnicities living along the Thailand-Myanmar border. *Wellcome Open Research*.2017;2: 10.12688/wellcomeopenres.12338.1 10.12688/wellcomeopenres.12338.1 29181452PMC5686509

# Response

**Published:** 2017-10-23

**Authors:** Germana Bancone

**Affiliations:** Shoklo Malaria Research Unit, Thailand

We thank the reviewer for his useful comments and suggestions. Below the answers to his questions.

**Major comments**

 Q1) The background to the study is rationalized on the basis of the potential importance of inherited red cell disorders as causes and confounders of anaemia in pregnant women within the region.  The inclusion of data on ABO and Rhesus in this paper, though interesting, does not fit well within this rational framework.  Neither are particularly relevant to the main story. I would either drop those data or alter the rationale to justify their inclusion. 

 A1)Although the description of ABO and Rhesus blood groups was meant to add information on the genetic background of ethnic groups, we agree with the reviewer that it does not fit well with the framework of the manuscript. We have therefore decided to drop this part.

 Q2) The referencing could be improved throughout the introduction. For example, a reference for Lyonisation and G6PD activity could be added, and the third paragraph, which contains a lot of technical information, should be referenced along the way rather than through the bunching of 4 (slightly random) references at the end.

 A2) We have added more references in the introduction. The 4 references mentioned by the reviewer only concerned the selection by malaria of haemoglobinopathies.

 Q3) Introduction paragraph 3 could be tightened up. The first sentence is a bit confused: “..structural variants and deletional mutations on the hemoglobin …. chains” is a bit of a mixture of nomenclature more usually specific to either the protein or the gene.  The “deletional mutations” more commonly refer to the gene than the protein and probably more correctly should be referred to as “genetic deletions” rather than mutations (which more commonly relate to sequence changes than deletions).  Further down, “…97% of hemoglobin is composed of two alpha and two beta chains” – the authors should clarify that they’re talking about ADULT haemoglobin (HbA). The references on the geographic distribution with malaria are a bit random and refer to different IRDs. A recent authoritative review would probably be a better way to go.

 A3) We have reviewed and corrected the paragraph to include the reviewer’s suggestions. 

 Q4) Methods: Lab analyses. It would be very helpful if the authors explained here on behalf of the non-expert what the G6PD spot test is designed to do and at what level of normal G6PD activity the test turns positive. A major issue with this test, as the authors acknowledge, is that it is a binary test that shows sufficient / deficient only. It is not designed to pick up heterozygotes and is only partially sensitive for homozygotes, depending on the genotype involved and the degree to which it perturbs G6PD production.  Some discussion of this in the methods section would help the non-expert reader.

 A4) We have revised the text to better explain the G6PD fluorescent spot test.

 Q5)  In the same section, the authors state that the quantitative test was conducted in “selected women”. It took me some digging to find that it was actually conducted in “a sub-set of sequentially recruited [and therefore “unselected”] women”. This could be better phrased at this point.

 A5) We agree with the reviewer that this part of the method was not phrased clearly and we have modified it.

 Q6) It would help the reader if the flow chart (Figure 1) could be amended to make the above more clear with regard to the quantitative test and the genotyping for both G6PD and other conditions. 

 A6) The Flow chart has been modified to exclude results of ABO and Rh blood groups and to clarify G6PD and Hb genotyping.

 Q7)  Methods; haemobloginopathies.  The authors used an HbA2 of <2.2 or <2.1% (depending on the method) to define “presumptive” carrier status for alpha-thalassaemia.  As the authors state later in the paper, this is neither a sensitive nor specific approach to the diagnosis of the alpha-thalassaemias overall. It’s almost useless for the carrier forms of the common minor single gene -3.7 and -4.2kb deletional forms of the condition.  As such, the authors’ focus on this sub-group for further work up for alpha thalassaemias constitutes a biased sample.  It would really have been better from the perspective of trying to determine the prevalence of these conditions if the authors had centred their genetic testing on an unselected sample of women.  This is the approach they took for G6PD genotyping which therefore produced figures that were more understandable. The figures given for the prevalence of the various alpha thalassaemias are difficult to interpret and probably very misleading because of the way in which the patients were sampled.

 A7) We thank the reviewer for pointing out this issue that we got wrong in the first version of the manuscript. At the time we started the screening we were not fully aware of the low specificity of the Hb typing for alpha-thalassaemia and one of our aims was to assess the diagnostic power of CE/HPLC for all hemoglobin variants. Nevertheless we agree that the way we presented the data was not appropriate and that the prevalence of alpha gene mutations in the subgroup of women with low HbA _2_ is not representative of the general population. We have therefore modified the text and the Table. This has also allowed correcting the numbers of women with low HbA _2_ tested by PCR which is 354 and not 374 as reported previously.

 Q8) For this reason, I would remove the rows regarding “suspected alpha thalasssaemia” from table 5 – the authors say later on that half of the sub-sample they tested were normal genetically, so this row will mislead the casual reader. HbCS and HbE are more legitimate as these have their own HPLC peaks.

 A8) This has been done.

 Q9) Discussion, paragraph 2 seems a bit odd and comes rather out of the blue. This is the first mention of anthropology that I can find in the paper and the use of these IRDs as anthropological tools is put up and shot down in a single sentence!

 A9) Since we have compared all these genetic markers between ethnic groups, one might argue that they could be used to estimate genetic differences between the ethnicities. We have removed this sentence together with the analysis of ABO/Rh blood groups.

 Q10) Authors could be more clear that their estimate of G6PDd on the basis of their spot test result will be an underestimate, since homozygosity for less severe variants will not always be picked up (as shown by the outlier on Fig 2).

 A10) We have added and clarified a few sentences to emphasize this point which is already well described in the literatures. With this analysis we wanted to concentrate the attention on the distribution of G6PD activities among heterozygous women which has not been studied extensively before.

 Q11) Discussion page 10 paragraph 2 left column. The authors say that all women with “Hb variants” are informed of the results and counseled that they may experience weakness etc in pregnancy.  I assume that this is not the case for “suspected alpha thalassaemia”? This would give rise to a great deal of unnecessary anxiety if half don’t actually have the condition anyway and in the case of the -3.7 and -4.2 deletions they are clinically silent when inherited alone.  The whole area of how to approach antenatal counseling in populations with a high prevalence of multiple conditions is highly complex and the discussion of this element could be tightened up a bit.  

  A11) It is very difficult to carry on a proper ante-natal screening and counseling in our setting and we are fully aware of the limitations of our approach. At the beginning all women diagnosed with abnormal Hb were counseled. As explained already in A7 the first aim for genotyping subjects with low %HbA _2_ was to rule out alpha thalassaemia carriage; when genotyping became available and the results showed that half of women did not have mutations on the alpha genes and another 40% were silent carriers, we modified the counseling accordingly.

 We are aware the discussion is quite long but we also wanted to touch on the several aspects that make this setting peculiar.

**Minor**

 1) Though it’s an editorial decision, I would have assumed that since this is a UK based journal the spelling should be in UK English throughout.

 We agree and have changed the spelling to UK English; more than an editorial choice, the first author is an Italian who has a relaxed approach towards spelling. 

 2) Abstract Methods: Blood groups were “diagnosed” makes it sound as if these are a disease! I would change to “determined” or similar.

 We have cut this part.

 3) “resource rich countries” – preferred current term is “high-income countries”

 This has been changed

 4) Final paragraph of the introduction could probably be condensed and moved to join the methods section. I would replace by a short sentence summarizing the aims of the current study

 This has been done.

 5) Please give the make and model of the HPLC machine used for the haemoglobin phenotyping.

 We have added it.

 Could do with a good proof-read throughout. A few exapmples I picked up:

 1) Introduction line 3 “…living in area of past…” should read “…living in areas of past…”

 2) Abstract Methods: Data were available rather than data was available (data are plural in this context).

  3) page 4 line one column 2 – “SAE” should read “SEA”.

 4) Table 6 – use the Greek symbol for alpha rather than the letter A.

 5) page paragraph 2 left column “found to be carrier..” should read “found to be carriers..”

 We have revised the whole manuscript and hopefully corrected all typos and incorrect terms.
